# Six novel Y chromosome genes in *Anopheles* mosquitoes discovered by independently sequencing males and females

**DOI:** 10.1186/1471-2164-14-273

**Published:** 2013-04-23

**Authors:** Andrew Brantley Hall, Yumin Qi, Vladimir Timoshevskiy, Maria V Sharakhova, Igor V Sharakhov, Zhijian Tu

**Affiliations:** 1 Department of Biochemistry, Virginia Tech, Blacksburg, VA, USA; 2 Department of Entomology, Virginia Tech, Blacksburg, VA, USA

## Abstract

**Background:**

Y chromosomes are responsible for the initiation of male development, male fertility, and other male-related functions in diverse species. However, Y genes are rarely characterized outside a few model species due to the arduous nature of studying the repeat-rich Y.

**Results:**

The chromosome quotient (CQ) is a novel approach to systematically discover Y chromosome genes. In the CQ method, genomic DNA from males and females is sequenced independently and aligned to candidate reference sequences. The female to male ratio of the number of alignments to a reference sequence, a parameter called the chromosome quotient (CQ), is used to determine whether the sequence is Y-linked. Using the CQ method, we successfully identified known Y sequences from *Homo sapiens* and *Drosophila melanogaster.* The CQ method facilitated the discovery of Y chromosome sequences from the malaria mosquitoes *Anopheles stephensi* and *An. gambiae.* Comparisons to transcriptome sequence data with blastn led to the discovery of six *Anopheles* Y genes, three from each species. All six genes are expressed in the early embryo. Two of the three *An. stephensi* Y genes were recently acquired from the autosomes or the X. Although *An. stephensi* and *An. gambiae* belong to the same subgenus, we found no evidence of Y genes shared between the species.

**Conclusions:**

The CQ method can reliably identify Y chromosome sequences using the ratio of alignments from male and female sequence data. The CQ method is widely applicable to species with fragmented genome assemblies produced from next-generation sequencing data. Analysis of the six Y genes characterized in this study indicates rapid Y chromosome evolution between *An. stephensi* and *An. gambiae*. The *Anopheles* Y genes discovered by the CQ method provide unique markers for population and phylogenetic analysis, and opportunities for novel mosquito control measures through the manipulation of sexual dimorphism and fertility.

## Background

Originating independently in diverse taxa, the XX/XY sex determination system is a striking example of convergent evolution [1-5]. In many species with Y chromosomes, a Y-linked dominant male-determining gene is hypothesized to initiate male sexual differentiation [2,6,7]. However, Y-linked dominant male-determining genes have eluded characterization with only a few exceptions. In therian mammals the dominant male-determining factor, *SRY*, is one of the few Y-linked genes retained after the degeneration of the Y chromosome [8-11]. In the medaka fish *Oryzias latipes* the Y-linked gene *DMY* functions as the dominant male determining gene [7].

Numerous insect species determine sex through the XX/XY sex-determination system, although insect Y chromosomes likely originated independently multiple times from diverse origins [1-3,12]. A notable exception is that while *Drosophila* species have Y chromosomes, sex is not determined by a gene on the Y, but instead the X-to-autosome ratio [13]. Although no Y-linked sex-determining genes have been characterized in insects, Y-linked sex-determining genes likely exist in *Anopheles* mosquitoes, the housefly *Musca domestica*, the Mediterranean fruit fly *Ceratitis capitata*, and possibly many more species with Y chromosomes [3,14,15]. Most insect Y chromosomes remain entirely unexplored.

Over time, male-beneficial genes may accumulate on the Y chromosome by gene duplications [16]. For example, all known protein-coding genes on the *D. melanogaster* Y chromosome originated from autosome-to-Y duplications [16-21]. Due to their exclusive presence in males, Y chromosomes may also serve as a sanctuary for genes advantageous to males, and deleterious to females [22].

Y chromosome genes have eluded all but the most determined attempts at characterization due to the heterochromatic and repetitive nature of the Y [19,23]. Using bacterial artificial chromosomes (BACs) and an iterative mapping and sequencing strategy, the sequence of the euchromatic region of the Y chromosome was obtained for humans and two other primates [9,10]. The iterative mapping strategy used in primates has not been implemented to sequence other Y chromosomes due to the cost associated with the technique. In insects, the sequences of Y genes have only been characterized in a few *Drosophila* species. The *Drosophila* Y gene sequences were found by a strategy, developed by Carvalho and colleagues, based on the comparison of sequences not anchored to chromosomes to known protein sequences [17,19]. This method may be difficult to implement in species without a reference genome where most of the sequences are anchored to chromosomes, and it failed to identify any Y genes in the African malaria mosquito *An. gambiae*[24].

In this study, we introduce the chromosome quotient (CQ) method, a novel approach to systematically discover Y genes. The CQ method takes advantage of high-throughput genome and transcriptome sequencing data and does not require a reference genome where most of the sequences are anchored to chromosomes. We used the CQ method to find Y sequences from two important malaria mosquitoes, *An. stephensi* and *An.* gambiae, members of the same subgenus *Cellia*[25]. Comparison of the Y sequences to transcriptome sequence data led to the discovery and characterization of six *Anopheles* Y genes, three from each species. Evolutionary analysis strongly suggests rapid Y chromosome evolution in *Anopheles* mosquitoes.

## Results

### The chromosome quotient (CQ) method

We developed the CQ method based on the following observations. Y chromosomes are present only in males. Therefore, sequences unique to the Y should be present in male sequence data and absent from female sequence data. Using this principal, we developed a method to identify Y sequences by comparing separate male and female sequence data to a reference genome. Unique Y sequences can be identified because they are only present in the male sequence data. However, searching for sequences exclusive to the male sequence data yielded only a few short sequences because most Y sequences appear to be at least partially repetitive. We realized that if the reference genome was fragmented into smaller pieces by removing repeats, more sequences exclusive to the male sequence data could be identified.

Y chromosomes appear to be primarily composed of repetitive sequences that are not exclusive to the Y. Since most Y-repeats have closely-related sequences on the autosome or X, they may appear be present in both the female and male sequence data. Recent duplications of autosome or X sequences to the Y may also cause Y sequences to appear to be present in both the male and female sequence data. We reduced the interference from repetitive sequences by using very strict alignment criteria. We require zero mismatches over the entire length of the reads from the male and female sequence data. We increased the number of Y chromosome sequences identified by allowing a few alignments from the female sequence data, as long as there were many more alignments from the male sequence data.

To define the number of alignments allowed from female data we use the ratio of female to male alignments, a parameter we call the chromosome quotient (CQ). For a given sequence S_i_, CQ_(Si)_ = F_(Si)_/M_(Si)_, where F_(Si)_ is the number of alignments from female sequence data to S_i_, and M_(Si)_ is the number of alignments from male sequence data to S_i_. The method by which chromosome quotients are calculated is found in the additional files (Additional file 1: Figure S1).

The chromosome quotient allows for the differentiation of Y sequences from autosome and X sequences (Additional file 2: Figure S2). Males and females share the same complement of autosomes, so autosomal sequences are present in both male and female sequence data in roughly the same quantities. Therefore, autosomal sequences have chromosome quotients distributed around one. Females have two X chromosomes while males have only one, so X chromosome sequences are present in female sequence data roughly twice as frequently as in male sequence data. Therefore, X chromosome sequences have chromosome quotients distributed around two. Unique Y sequences are present only in male sequence data, and therefore have chromosome quotients of zero. Repetitive Y sequences are present in both the male and female sequence data and have chromosome quotients greater than zero but less than the chromosome quotients of autosomal sequences. We set a threshold of CQ = 0.3 to differentiate Y sequences from autosome and X sequences.

Using the CQ method, a sequence is classified as Y-linked if it has more than 3.33 times as many alignments from male data than from female data, and therefore a CQ less than 0.3. In case the coverage of the male and female sequence data differs, chromosome quotients are normalized to the median chromosome quotient of known autosomal sequences.

### Y chromosome sequences have distinctive chromosome quotients

**Figure 1:**
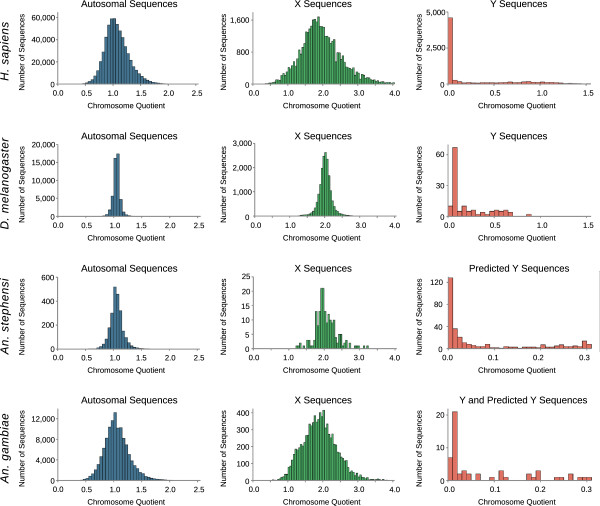
**Y chromosome sequences have distinctive chromosome quotients.** The distribution of chromosome quotients calculated for autosome, and X sequences from *H. sapiens*, *D. melanogaster*, *An. stephensi*, and *An. gambiae,* the distribution of chromosome quotients for Y sequences from *H. sapiens*, and *D. melanogaster*, the distribution of chromosome quotients from predicted Y sequences from *An. stephensi*, and the distribution of chromosome quotients from known and predicted Y sequences from *An. gambiae*. The predicted Y chromosome sequences from *An. stephensi* originated from the contigs of the 454 genome assembly. The predicted Y chromosome sequences from *An. gambiae* originated from the UNKN sequences and our own Illumina assembly.

To validate that the chromosome quotients of Y sequences are distinctive from those of autosome and X sequences, the CQ method was tested in *H. sapiens* and *D. melanogaster*. Autosome, X, and Y sequences were downloaded for both *H. sapiens* and *D. melanogaster.* The repetitive sequences indicated by RepeatMasker were removed, fragmenting the genomes into many smaller pieces. Male and female sequence data was located for both species. The chromosome quotients of the fragmented autosome, X, and Y sequences were calculated using the separate male and female sequence data (Figure 1, Table 1).

**Table 1:** The number of sequences and the normalized CQs of the positive control data

**Data set**	**Autosomal sequences**	**Normalized median CQ**	**X sequences**	**Normalized median CQ**	**Y sequences**	**Normalized median CQ**
*H. sapiens*	581,737	1.00	39,844	1.85	7,934	0.00
*D. melanogaster*	48,870	1.00	18,011	1.96	139	0.025

The number of sequences and the normalized median chromosome quotients (CQs) of the positive control data sets illustrate that autosomal sequences have chromosome quotients distributed around one, X sequences have chromosome quotients distributed around two, and Y sequences have chromosome quotients near zero. The distributions of chromosome quotients from different chromosomes are shown in Figure 1.

In both species, autosomal sequences have chromosome quotients distributed around one, X sequences have chromosome quotients distributed around two, and most Y sequences have chromosome quotients near zero. The median chromosome quotients of autosome and X sequences are significantly greater than the median chromosome quotients of Y sequences (Mann Whitney *U* Test p = 0 for X-to-Y and auto-to-Y comparisons in both species). Thus, Y sequences can be identified by their distinctive chromosome quotients. The interval of chromosome quotients (0.0, 0.3) captures more than 67 percent of the known Y sequences with a rate of false positives less than 2.5 percent in *H. sapiens* and *D. melanogaster* (Table 2). Therefore, we chose this threshold for the discovery of Y chromosome sequences in the mosquitoes *An. stephensi* and *An. gambiae.*

**Table 2:** The false positive and false negative rates of the CQ method

**Data set**	**Total autosome and X sequences**	**Autosome or X sequences with CQ < 0.3**	**False positive rate**	**Total Y sequences**	**Y sequences with CQ <0.3**	**False negative rate**
*H. sapiens*	621,581	135	2.44%	7,934	5,408	31.8%
*D. melanogaster*	66,881	2	1.85%	139	106	23.7%

The false positive rate is highly dependent on the CQ cut-off below which a sequence is classified as Y-linked. In this study, we used a CQ cut-off of 0.3 as it minimized false positives while keeping false negatives relatively low. There is flexibility in selecting the CQ cut-off according to the focus of the study and the scale of downstream verification that the investigators plan to perform.

### Discovery of novel Y sequences in *Anopheles* mosquitoes

We used the CQ method to discover novel Y sequences in two malaria mosquitoes. In this study, we consider a sequence to be Y-linked if it has a chromosome quotient less than 0.3, meaning it has more than 3.33 times more alignments from male data than from female data.

**Figure 2:**
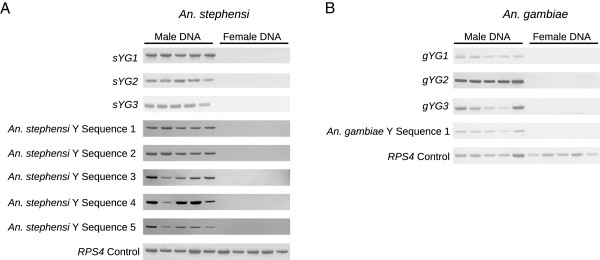
**Male-specific PCR amplification of Y genes and intergenic Y sequences.** (**A**) PCR performed with five male and five female genomic DNA samples shows male-specific amplification of three *An. stephensi* Y genes along with five *An. stephensi* intergenic Y sequences. A *RPS4* ribosomal protein gene was amplified in both male and female genomic DNA confirming the integrity of the genomic DNA samples. (**B**) PCR performed with five male and five female genomic DNA samples shows male-specific amplification of the three *An. gambiae* Y genes, along with an intergenic *An. gambiae* Y sequence. A *RPS4* ribosomal protein gene was amplified in both male and female genomic DNA confirming the integrity of the genomic DNA samples.

We first searched for Y sequences in the Asian malaria mosquito *An. stephensi*, a species with a draft genome generated by 454 sequencing [GenBank: ALPR00000000]. We performed Illumina sequencing on male and female *An. stephensi* genomic DNA [SRA: SRP013838]. Chromosome quotients were first calculated for all known autosome and X contigs in the *An. stephensi* genome (Figure 1). All of the autosome and X sequences had chromosome quotients greater than 0.3. Chromosome quotients were then calculated for 113,570 contigs from the *An. stephensi* genome. We identified 317 candidate Y sequences with chromosome quotients less than 0.3, encompassing more than 130,000 bases (Figure 1). Five of these sequences were tested for male-specific amplification with PCR on male and virgin-female genomic DNA (Table 3). All five sequences amplified a product exclusively in male genomic DNA (Figure 2).

**Table 3:** The chromosome quotients of sequences tested for Y-linkage with PCR

**Sequence name**	**Number of female alignments**	**Number of male alignments**	**Normalized chromosome quotient**
*sYG1*	1	553	0.002
*sYG2*	0	29	0.000
*sYG3*	1	158	0.005
*gYG1*	3	1631	0.002
*gYG2*	2	692	0.003
*gYG3*	19	340	0.056
*An. stephensi* Y sequence 1	0	211	0.000
*An. stephensi* Y sequence 2	1	305	0.003
*An. stephensi* Y sequence 3	4	148	0.023
*An. stephensi* Y sequence 4	10	335	0.026
*An. stephensi* Y sequence 5	2	61	0.029
*An. gambiae* Y sequence 1	13	101	0.129

All of the sequences listed amplified a PCR product exclusively in male genomic DNA, and all have chromosome quotients less than 0.3. We made an exception for *sYG2* as it only has 29 alignments from male data, and the cutoff was 30 (see Methods). Even at lower stringency, *sYG2* still had no female alignments so we decided to include it. The chromosome quotients for *gYG1* and *gYG2* were calculated from the transcripts because of the long introns present in the genes.

We then applied the CQ method to the genome of the African malaria mosquito, *An. gambiae.* We performed Illumina sequencing on male and female *An. gambiae* genomic DNA [SRA: SRP014730]. The *An. gambiae* AgamP3 genome assembly was downloaded from VectorBase. Repetitive sequences identified by RepeatMasker were removed, fragmenting the genome into many smaller pieces. Chromosome quotients were calculated for the fragmented autosome and X sequences from the AgamP3 genome assembly (Figure 1). The autosomal sequences had chromosome quotients distributed around one, and the X chromosome sequences had chromosome quotients distributed around two. Chromosome quotients were calculated for the fragmented Y chromosome sequences from the AgamP3 genome assembly. Of the 42 known *An. gambiae* Y sequences, 37 had chromosome quotients less than 0.3.

The *An. gambiae* sequences that were unable to be anchored to chromosomes, known as UNKN in the AgamP3 assembly, were fragmented by removing the repeats indicated by RepeatMasker. Chromosome quotients were calculated for these UNKN sequences. From the UNKN sequences, we identified 16 novel candidate Y sequences, encompassing more than 9,000 bases (Figure 1).

We also tested the CQ method *de novo* by generating a genome assembly from male G3 strain Illumina sequence data using ABySS. Chromosome quotients were calculated for all the sequences in the Illumina assembly. From the Illumina assembly, we identified 17 novel candidate Y sequences encompassing more than 6,000 bases (Figure 1). All Y sequences reported in this study are provided in the additional files (Additional file 3).

### Discovery and expression profile of six *Anopheles* Y genes

**Figure 3:**
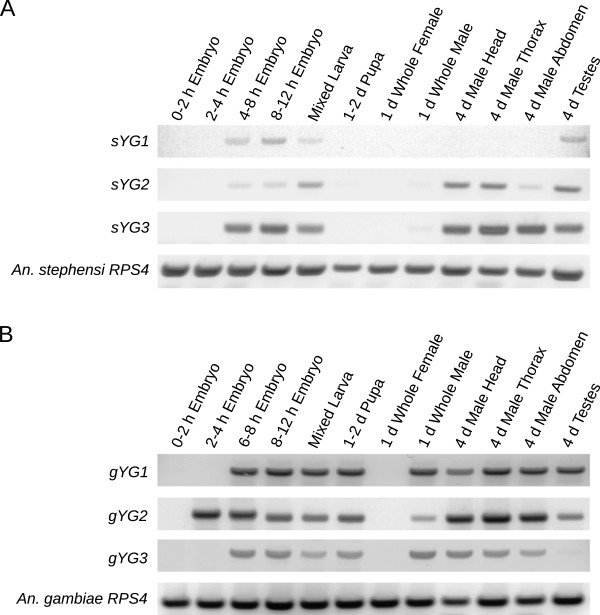
**Expression profile of six Anopheles Y genes.** (**A**) The expression profile of the three *An. stephensi* Y genes sYG1, sYG2, and sYG3. (**B**) The expression profile of the three *An. gambiae* Y genes gYG1, gYG2, and gYG3. For both species, a ribosomal protein gene (RPS4) was used as positive control. RT-PCR was performed with primer-sets that demonstrated male-specific amplification in PCR on genomic DNA (Additional file 1: Table S1). The expression profile was performed on time points spanning from 0–2 hour embryos to adults. Mixed larvae refers to mixed L1 to L4 instars; h, hour; d, day.

**Figure 4:**
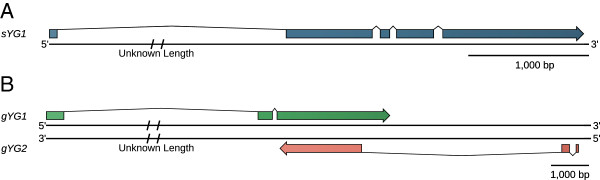
**The structure of *sYG1*, *gYG1*, and *gYG2 *as determined by comparing cDNA and genome sequences.** (**A**) The structure of *sYG1*. The first intron of *sYG1* is likely very long as the start of the 5′ RACE aligns to a different scaffold in the draft *An. stephensi* genome. (**B**) *gYG1* and *gYG2* are overlapping genes that encode distinct RNAs from opposite strands.

Comparison of the Y sequences to *An. stephensi* and *An. gambiae* RNA-sequencing data with blastn led to the identification of six novel Y genes, three from each species (Table 3). The *An. stephensi* and *An. gambiae* Y genes were given the names *sYG* (*An. stephensi Y gene*) and *gYG (An. gambiae Y gene*) respectively, followed by a number designating the order in which they were discovered. All six Y genes amplified a product exclusively in male genomic DNA (Figure 2). Sequencing of RT-PCR products (Figure 3) confirmed transcription of all six genes. cDNA sequences for genes with confirmed introns were used to determine the structure of these genes, and are provided in Additional file 3 (Figure 4). Interestingly, all six genes were expressed in the early embryo, 2–12 hours after egg deposition (Figure 3). None of the six genes were expressed in 0–1 hour embryos, when only maternal transcripts are present, or in the adult female where no Y chromosome is present [26,27].

### The three *An. stephensi* Y genes include two recent duplications to the Y and a novel Y gene

**Figure 5:**
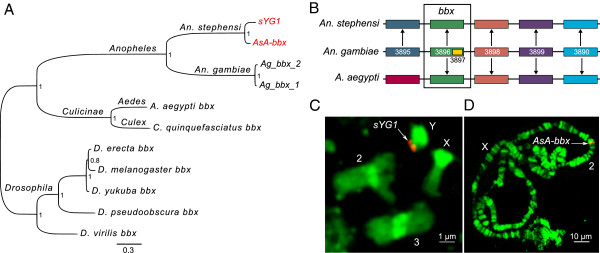
***sYG1 *is the result of a recent autosome-to-Y duplication.** (**A**) Phylogenetic analysis of *bobby sox* (*bbx*) genes from different species. Alignments were produced by Clustalw. Phylogenetic inference was performed using MrBayes v3.1.2. The number near each node is the credibility score provided by MrBayes. Note that *Ag_bbx_1* and *Ag_bbx_2* are the two *An. gambiae* autosomal genes *AGAP003986* and *AGAP003987*, respectively. (**B**) Synteny of the genes located around *bobby sox* is largely maintained in *An. gambiae*, *An. stephensi*, and *Aedes aegypti*. Genes indicated by the same color are orthologs. The *An. gambiae* gene names are preceded by *AGAP00*, the VectorBase gene designations. *An. gambiae* gene *AGAP003897* is in an intron of *AGAP003896*. *A. aegypti* orthologs were assigned by VectorBase. *An. stephensi* orthologs were assigned by our own genome annotation. (**C**) FISH with a probe designed according to *sYG1* hybridized to the *An. stephensi* Y chromosome. Mitotic chromosome slide preparations were prepared from imaginal discs of 4th instar larvae. Mitotic chromosome FISH was used because the *An. stephensi* Y chromosome does not polytenize in salivary glands and the ovarian polytene chromosomes do not contain a Y chromosome. (**D**) The autosomal copy of *bobby sox* in *An. stephensi*, *AsA-bbx*, was mapped to chromosome 2R with FISH on polytene chromosomes. Polytene chromosome slide preparations were obtained using ovaries of half gravid females. From these results we can deduce that *AsA-bbx* is the ancestral copy of *bobby sox* in *An. stephensi*, and that *sYG1* is the result of a recent duplication from chromosome 2R to the Y.

**Figure 6:**
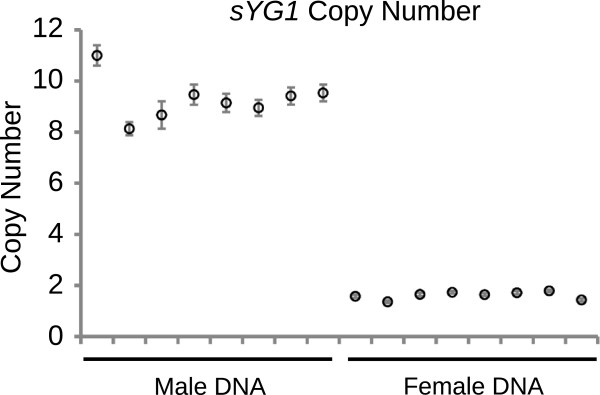
***sYG1 *is a multi-copy gene on the Y.** Copy number determination using digital droplet PCR designed to detect both *sYG1* and *AsA-bbx* indicates two autosomal *AsA-bbx* copies (seen in females) and six-to-nine copies of *sYG1* in males (8 to 11 copies minus the two autosomal copies). Since we know that *sYG1* hybridizes to the Y in FISH, we can infer that the extra copies of *sYG1* in males is due to the fact that there are many copies of *sYG1* on the Y. Digital PCR partitions a sample into as many as 20,000 individual droplets and uses TaqMan chemistry with fluorescently labelled probes to accurately determine the copy-number of genes. An autosomal reference gene (sequence provided in Additional file 3) was set as having two copies per diploid genome. The copy number of *sYG1*/*AsA-bbx* was determined by comparison to the autosomal reference. Error bars represent poisson error.

*sYG1* [GenBank: KC822952] shares 95 percent nucleotide identity with a gene mapped to chromosome 2R (Table 4, Figure 5). We call this autosomal paralog of *sYG1, AsA-bbx* (*An. stephensi autosomal bobby sox*). Both *sYG1* and *AsA-bbx* are homologous to the *D. melanogaster* gene *bobby sox* (*bbx*) and to two closely linked *An. gambiae* genes, *AGAP003896* and *AGAP003897,* which are located on the *An. gambiae* 2R chromosome arm. *AsA-bbx* is part of a large contig and the synteny of its neighboring genes is conserved among *Anopheles* and *Aedes* mosquitoes (Figure 5) suggesting that *AsA-bbx* is the ancestral copy, and *sYG1* was formed from a recent duplication of *AsA-bbx* to the Y. Phylogenetic analysis suggests that this duplication of *sYG1* occurred after the evolutionary divergence of *An. stephensi* and *An. gambiae* (Figure 5). 5′ and 3′ RACE was performed on *sYG1* to characterize its full-length cDNA (Figure 4). Copy number determination using digital droplet PCR [28], designed to detect both *sYG1* and *AsA-bbx,* indicates two autosomal *AsA-bbx* copies per diploid genome (a single copy per haploid genome) and six-to-nine copies of *sYG1* in males (Figure 6). Chromosomal fluorescence *in situ* hybridization (FISH) with probes designed according to *sYG1* hybridized to the *An. stephensi* Y on male mitotic chromosomes and on the 2R arm on female polytene chromosomes (Figure 5). Similar to the digital PCR analysis, this FISH result confirms that *AsA-bbx* is a single-copy autosomal gene located in subdivision 18A of the 2R arm, while *sYG1* is a multi-copy gene on the Y.

**Table 4:** Five of the six Y genes share high nucleotide identities to non-Y sequences

**Y gene **^**1**^	**Non-Y paralog**	**Identity (E-value) **^**2**^
*sYG1*	*AsA-bbx*^*3*^	95% (0)
*sYG2*	None ^4^	Not applicable
*sYG3*	*An. stephensi AGAP000048*^5^	94.8% (0)
*gYG1*	3 L:34084227–34084914 ^6^	94% (0)
*gYG2*	3 L:34084227–34084914 ^7^	94% (0)
*gYG3*	2 L: 5229785–5230624 ^8^	89% (0)

The most similar non-Y sequences to the Y genes in the *An. stephensi* and *An. gambiae* genomes. 1. Query sequences are the nucleotide sequences of the Y genes shown in Additional file 3. 2. blastn is used to obtain e-values. Percent identities reflect the entire sequence except for *gYG1* and *gYG2*. 3. *AsA-bbx* is orthologous to *An. gambiae AGAP003896/7*. *AsA-bbx* is an autosomal paralog of *sYG1*. 4. *sYG2* has no similar sequence in the rest of the *An. stephensi* genome except for a 55 bp repetitive region. 5. *sYG3* is closely related to an annotated gene in *An. stephensi*, which is orthologous to *An. gambiae AGAP000048*. 6. *gYG1* appears to be a composite of different fragments that share high similarities to different un-annotated non-Y sequences in *An. gambiae*. Some fragments are repeated in the genome. Shown in this table is a 688 base fragment that is 94 percent identical to sequences in chromosome 2R, 3 L as well as unmapped scaffolds. 7. *gYG*2 and *gYG1* overlap, which is why the best non-Y match is identical between the two Y genes. *gYG*2 and *gYG1* are transcribed from opposite strands. *8. gYG*3 is most similar to an un-annotated 2 L sequence and an unmapped sequence in *An. gambiae*.

With the exception of a 55 base repetitive region, *sYG2* [GenBank: KC822953] matched no other sequences in the *An. stephensi* genome using blastn or tblastx with an e-value threshold of 1e-2. *sYG2* has no homolog in *An. gambiae* as indicated by blastn and tblastx searches of the PEST, M, and S genome assemblies as well as the trace files.

s*YG3* [GenBank: KC840348] shares 94.8 percent nucleotide identity with an autosomal or X gene within the 355,000 base scaffold00149 of the *An. stephensi* genome. This gene is homologous to *AGAP000048* on the *An. gambiae* X chromosome (Tables 4 and 5). *AGAP000048* and its neighboring genes showed conserved synteny in *An. gambiae*, *An. stephensi*, and *Culex quinquefasciatus*, suggesting that the autosomal or X paralog of *sYG3* is ancestral. Thus, we deduce *sYG3* is also a recent duplication to the *An. stephensi* Y that occurred after the divergence of *An. stephensi* and *An.* gambiae.

**Table 5:** Y gene homologs

**Y gene**	**Homolog**^**1**^	**Maximum percent identity**^**2**^	**E-value **^**2**^	**Homolog putative function**
*sYG1*	*AGAP003896*	35%	1e-39	*Bobby sox* (*bbx*) HMG-box transcription factor
*sYG2*	No homology found	-	-	-
*sYG3*	*AGAP000048*	37%	2e-13	Adenomatous polyposis coli protein
*gYG1*	*AGAP005574*	47%	1e-16	Unknown
	*AGAP011774*	36%	9e-08	Unknown
	*AGAP001079*^*3*^	89% ^3^	0.034 ^3^	Unknown
*gYG2*	*AGAP011734*^*4*^	38% ^4^	1e-5 ^4^	Unknown
*gYG3*	*AGAP012527*	33%	2e-26	General transcription factor II repeat domain

The annotated homologs of the Y genes provide clues to putative functions of the Y genes we discovered. 1. Homologs were identified using blastx against the *An. gambiae* protein database (AgamP3.6 Gene Build) and the NCBI non-redundant protein database. Not surprisingly, the best matches are to *An. gambiae* sequences because the queries are from *An. gambiae* and *An. stephensi*. The *An. gambiae* genes *AGAP003896* and *AGAP000048* are orthologous to the autosomal or X paralogs of *sYG1* and *sYG3*, respectively. The other homologs listed in column 2 are either distantly related or only share partial overlap. In cases where the Y genes match more than one related homolog, the homolog with the best e-value is shown. The three homologs shown for *gYG1* are unrelated. 2. Maximum percent identities and e-values were obtained by blastx against the *An. gambiae* protein database (AgamP3.6 Gene Build). For Y genes that have confirmed introns (*sYG1*, *gYG1*, and *gYG2*), transcript sequences were used as the query. 3. The match to *AGAP001079* is very short at the amino acid level although the nucleotide identity is 92 percent, and 98 percent in two longer fragments (e-value of 6×10^-18^). The sense strand of *gYG1* is reverse-complementary to *AGAP001079*. 4. The sense strand of *gYG2* is reverse-complementary to *AGAP011734*.

### The three *An. gambiae* Y genes have closely related autosomal or X paralogs in the *An. gambiae* genome

*gYG1* [GenBank: KC840350] and *gYG2* [GenBank: KC845524] are two new genes found in the Y chromosome sequences of the AgamP3 genome assembly. Although they overlap on opposite strands, there are unique transcribed regions that allowed specific RT-PCR products to be amplified for each gene (Figures 3, 4). Both genes are highly transcribed from the early embryo until the adult male, including in the testes. Sequencing of RACE and RT-PCR products confirmed the organization of these two genes (Figure 4). Both genes have closely-related autosomal or X paralogs although these paralogs are not annotated (Table 4). Homologs of *gYG1* and *gYG2* were not identified in *An. stephensi* by blastn and tblastx searches of the genome assemblies as well as the 454 trace files, although a short repetitive region of *gYG1* does have significant alignments to the *An. stephensi* genome from tblastx.

A portion of the *gYG1* transcript shares more than 92 percent nucleotide identity with the *AGAP001079* group of genes on both the X and autosomes, while the flanking regions are distantly but significantly related to *AGAP005574* and *AGAP011774,* respectively. A portion of the *gYG2* transcript shares greater than 90 percent nucleotide identity with a multi-copy non-coding sequence in the *An. gambiae* genome. Another part of *gYG2* also contains a region that is homologous to *AGAP011734* (Table 5). The *gYG1* and *gYG2* transcripts are reverse complementary to *AGAP001079* and *AGAP011734*, respectively. This transcription orientation coupled with the fact that *gYG1* and *gYG2* are in opposite orientation may indicate functions related to double-stranded RNA formation, rather than coding for proteins. Because there are no known orthologs to *AGAP001079, AGAP005574,* and *AGAP011774* we could not determine the ancestry of the *gYG1* and *gYG2* genes relative to their paralogs.

*gYG3* [GenBank: KC840349] was discovered in an assembly of our own *An. gambiae* Illumina data. There are two similar sequences in the *An. gambiae* genome, both sharing 89 percent nucleotide identity with *gYG3* (Table 4). One is on chromosome 2 L and the other is not mapped but its chromosome quotient suggests it is an autosomal or X sequence. Thus, *gYG3* is again involved in a recent duplication, although we cannot assign the direction of the duplication. A blastx search shows that *gYG3* is distantly related to *AGAP012527* (OrthoDB Group EOG5VHHNX), which belongs to a large family of general transcription factor II-I repeat domain-containing proteins (Table 5).

## Discussion

Here we have shown that the CQ method can reliably identify Y sequences using the ratio of alignments from male and female sequence data. Using the CQ method we identified 350 Y chromosome sequences encompassing more than 145,000 bases of novel Y chromosome sequences from the malaria mosquitoes *An. stephensi* and *An. gambiae*.

It is also possible to identify Y sequences by coverage depth [25]. In male sequence data, Y sequences should have half the coverage of autosomal sequences. In mixed male and female sequence data, Y sequences should have one-quarter the coverage of autosomal sequences [29]. The CQ method does not directly rely on coverage depth to identify Y sequences. While Y sequences often do have low coverage depth, it is often not sufficient to classify a sequence as Y-linked solely by low coverage depth. Mis-assemblies of the reference genome, and allelic differences between the reference genome and the short-read sequence data could lead to low coverage depth causing errant classification of Y-linkage. Unmasked repetitive sequences may lead to high coverage depth, obfuscating many Y sequences. Instead, the CQ method uses the ratio of female to male alignments to a reference sequence. The CQ method takes into account both the lack of female alignments and the presence of male alignments when classifying sequences as Y-linked, reducing the overall rate of false positives and false negatives. Mis-assemblies and allelic variation can be detected by the lack of male reads aligned to a sequence. For a sequence to be classified as Y-linked with the chromosome quotient it must have many alignments from males, and zero or a few alignments from females.

Although the functions of the Y genes that we found are not yet clear, there are some tantalizing hints to their potential functions. *sYG1* is homologous to an HMG-box transcription factor involved in a number of developmental functions. HMG-box transcription factors include the two key genes involved in male-determination in mammals [30,31]. *sYG3* shares homology with a general transcription factor II repeat domain containing protein, indicating the possibility that it is a Y-linked transcription factor. Five of the Y genes are clearly expressed in the testes suggesting that they may perform male-specific roles. All six Y genes are expressed in the early embryo, which is the time when sexual differentiation begins [26,27].

Our research offers an opportunity to investigate Y chromosome evolution, which until now has been restricted to limited lineages. Primate Y chromosomes have been shown to undergo strict conservation following rapid gene loss [9]. In *D. melanogaster,* however, almost all the Y genes are the result of duplications to the Y. Here we have shown that two of the three *An. stephensi* Y genes were recently acquired from the autosomes or the X. All three *An. gambiae* Y genes have highly similar paralogs on the autosomes or X (Table 4), again consistent with recent duplication. Thus, gene acquisitions through duplications appear to be a major source of Y genes in *Anopheles* mosquitoes. This is intriguing given that unlike *D. melanogaster*, the *Anopheles* Y is thought to harbor a dominant male-determining factor that initiates sexual differentiation, similar to humans and several non-*Drosophila* flies [6].

So far, no conservation between the genes on the two *Anopheles* Y chromosomes has been found, although the two species belong to the same subgenus. It is possible that we simply missed the common Y genes that originated prior to the divergence between *An. gambiae* and *An. stephensi*. However, such shared Y genes are less likely to have highly similar autosomal or X paralogs compared to recent duplications, and they may even be unique to the Y. Y genes that are unique or significantly diverged from their autosomal or X paralogs are the easiest to find with the CQ method. To mitigate the effect of potential underrepresentation of Y genes in the *Anopheles* genome sequences, we compared the trace sequence files of both species and found no indications of shared Y genes. Thus, the evidence so far suggests that the *An. gambiae* and *An. stephensi* Y chromosomes are very different in their gene content. This conclusion is consistent with the observation that two of the three Y genes in *An. stephensi* were recently acquired after the divergence of the two mosquitoes*.* Further investigation of Y genes in additional *Anopheles* species will reveal whether the evolutionary scenarios demonstrated by the *Drosophila* Y chromosomes (10), namely recent Y gene acquisition and Y chromosome replacement, are widely applicable in species with a dominant male-determining factor on the Y.

Some of the Y genes described in this study have unique Y chromosome sequences. Because of their exclusive paternal inheritance and general lack of recombination, these Y sequences could serve as powerful molecular markers for the investigation of population structure and incipient speciation, which are often relevant to vectorial capacity [32]. Only female mosquitoes bite and transmit pathogens. Genetic strategies to control mosquito-borne diseases may include the release of sterile males (Sterile Insect Technique, SIT), the release of insects carrying a dominant lethal gene (RIDL), or the replacement of a pathogen-susceptible population with a pathogen-resistant one [33-37]. Under these scenarios, the release of males is either required or preferred. A better understanding of the possible functions of mosquito Y genes in sexual differentiation will facilitate the production of sterile males for improved SIT. It may also enable the production of highly competitive males to improve SIT and RIDL and to help spread pathogen resistance genes.

## Conclusions

In this study, we have shown that Y sequences can be identified by their distinctive chromosome quotients, the ratio of female to male alignments. Although the genomes of many organisms with Y chromosomes have been sequenced, Y sequences have been characterized from only a small subset of these genomes. Y sequences are surely present in these genomes, but are probably fragmented into short contigs challenging the traditional experimental methods of finding Y sequences. The quality of the genome assembly is not a limiting factor in finding Y sequences, making the CQ method widely applicable to species with fragmented genome. Autosome or X sequences rarely have chromosome quotients less than 0.3, giving the CQ method a low false positive rate. The CQ method is able to identify the majority of known Y sequences from species we have analyzed. We have also shown that the CQ method can identify recent duplications to the Y that share more than 94 percent nucleotide identity with autosomal or X paralogs. Coupling the CQ method with transcriptome sequencing allowed us to identify six Y genes in *An. stephensi* and *An. gambiae*. Analysis of the six Y genes indicates rapid Y chromosome evolution between *An. stephensi* and *An. gambiae*. The *Anopheles* Y genes characterized in this study provide unique markers for population and phylogenetic analysis, and opportunities for novel mosquito control measures through the manipulation of sexual dimorphism and fertility. The identification of Y genes in more organisms will help us understand the evolution and functions of Y chromosomes.

## Methods

### The CQ method

The chromosome quotient is the normalized ratio of female to male alignments to a given reference sequence. For a given sequence S_i_, *CQ*_(Si)_ = F_(Si)_/M_(Si)_, where F_(Si)_ is the number of alignments from female sequence data to S_i_, and M_(Si)_ is the number of alignments from male sequence data to S_i_.

To calculate chromosome quotients we wrote a program we call CQ-calculate (Available from: http://sourceforge.net/projects/cqcalculate/files/CQ-calculate.pl/download). CQ-calculate was written in Perl and designed to rapidly calculate chromosome quotients. There are three inputs to CQ-calculate: reference sequences, male-specific sequence data, and female-specific sequence data. The reference sequences are required to be in FASTA format. The male and female sequence data can be in either FASTQ or FASTA format. Preferably, the male and female sequence data should be from either highly inbred populations, or from a pool of many individuals to adequately sample genetic variation. For the best results, the male and female sequence data should be from the same colony or population to minimize the risk of bacterial or viral contamination exclusive to either the male or female data. The male and female sequence data is aligned to the reference sequences using the ultrafast read aligner Bowtie [38]. CQ-calculate uses stringent alignment criteria requiring the entire read to align with zero mismatches. To account for differences in coverage between the male and female sequence data, the chromosome quotients of the reference sequences are normalized to the median chromosome quotient of known autosomal sequences.

CQ-calculate can run on modest computers, and the only software requirements are: Linux, Perl, BioPerl, and bowtie. The time CQ-calculate takes to run is dependent on the genome size and coverage of the male and female sequence data. On a server running Ubuntu 12.04 with an Intel 3930 K six-core processor, it took approximately five minutes to calculate the chromosome quotients for all the sequences in the *An. stephensi* genome. For the much larger human genome, CQ-calculate took less than one hour. Memory requirements are dependent on the size of the reference genome but are typically minimal. CQ-calculate is easy to run, and is applicable to any heterogametic genome where separate male and female sequence data are available.

The CQ method uses the number of alignments from male and female sequence data to determine whether a sequence is Y-linked. The number of alignments affects the confidence with which a sequence can be classified as Y-linked. Thresholds were examined from one male alignment to 50 male alignments. Increasing the threshold for male alignments decreases the number of false positive results but also increases the number of false negatives (Additional file 4: Table S1). A threshold of 30 male alignments was chosen as it balances the number of false positives with the total number of sequences that can be analyzed. However, the threshold is flexible and can be increased for higher confidence in Y-linkage or decreased for a lower rate of false negatives.

We noticed that there are sequences with thousands of alignments from male and female data, but that still have many more male alignments than female alignments leading CQ-calculate to identify these sequences as Y-linked. Some of these sequences are known to be located on the autosomes or X, so we hypothesize that these are highly repetitive sequences with copies on the autosomes or X, but have many more copies on the Y. These highly repetitive sequences can be removed by setting a threshold for female alignments. In this study, we set a stringent threshold for female alignments of 30. Thus, to classify a sequence as Y-linked using the CQ method it must have a chromosome quotient less than 0.3, more than 30 alignments from male data, and less than 30 alignments from female data.

The coverage of the next-generation sequence data can affect the calculation of chromosome quotients. Since a threshold of alignments is required to classify a sequence as Y-linked, higher coverage male and female sequence data leads to the identification of more Y-sequences. We have tested a range of coverages and found that chromosome quotients can still be accurately calculated with as low as 5× coverage, but at this low coverage the number of Y sequences identified is reduced, and the false positive rate is increased. Above 10× coverage, many more Y sequences can be identified. With more than 20× coverage, many short Y sequences can be identified with a low rate of false positives.

### The CQ method positive control

We tested the CQ method on the genomes of *H. sapiens* and *D. melanogaster*. Since repeats can obscure Y sequences, we downloaded the softmasked reference genomes of *H. sapiens* (hg19 assembly) and *D. melanogaster* (dm3 assembly) from the USCS Genome Bioinformatics website. In a genome that is softmasked, repetitive sequences identified by RepeatMasker are replaced with lowercase nucleotides. We removed these repeats, fragmenting the genomes into smaller pieces. Fragments shorter than 250 bases were removed to mitigate false positives. The number of sequences resulting from the fragmentation, and the N50 size of the fragments were calculated (Additional file 5: Table S2).

*H. sapiens* male and female specific next-generation sequence data from a single male and female were downloaded from the 1000 Genomes Project [HG00234 and HG00235] [39]. Male and female *D. melanogaster* next-generation sequence data for pooled five day old mated adults was downloaded from the NCBI Sequence Read Archive [SRA: SRP007888]. The coverage of the *H. sapiens* and *D. melanogaster* data was calculated (Additional file 6: Table S3). Chromosome quotients were calculated for all the fragments of the *H. sapiens* and *D. melanogaster* genomes and then normalized to the median chromosome quotient of the known autosomal sequences.

### Y gene finding in the Asian malaria mosquito *An. Stephensi*

454 sequencing was performed on pools of approximately 30 adult *An. stephensi* from the Indian wild type strain. Newbler 2.6 was used to assemble the *An. stephensi* 454 sequence data into contigs and scaffolds. The contigs of this assembly are available from the NCBI [GenBank: ALPR00000000]. The number of contigs and N50 contig size were calculated (Additional file 5: Table S2). Pools of approximately 30 male and female *An. stephensi* of the Indian wild type strain were sequenced using the Illumina Genome Analyzer II [SRA: SRP013838] (Additional file 6: Table S3).

*An. stephensi* sequences have been anchored to chromosomes using chromosomal *in situ* hybridization [40]. The short probe sequences from FISH that hybridized to known autosome and X sequences were compared to the *An. stephensi* genome scaffolds using blastn requiring 95 percent identity. Autosome and X scaffolds were recovered. The scaffolds were then split into fragments by the ambiguous nucleotide N. Fragments shorter than 250 bases were removed. The chromosome quotients of these sequences were calculated.

Chromosome quotients were calculated for all the contigs from the *An. stephensi* genome using the male and female Illumina data mentioned above and normalized to the median chromosome quotient of autosomal sequences. Y genes were identified in the contigs with chromosome quotients less than 0.3 by comparison to transcriptome sequence data. Using blastn requiring 100 percent identity and an e-value less than 1×10^-5^ we compared the Y-linked contigs to transcriptome sequence data raw reads from *An.* stephensi Indian wild type strain. The time points compared were: 0–1 hour embryos, 2–4 hour embryos, 4–8 hour embryos, 8–12 hour embryos, mixed-instar larva, pupa, adult females, and adult males [SRA: SRP013839]. The number of reads for each time point was calculated (Additional file 6: Table S4).

We searched for evidence of the *An. stephensi* Y chromosome gene *sYG2* in the *An. gambiae* genome using blastn and tblastx. Blastn searches with a word size seven and e-value threshold of 1×10^-2^ using *sYG2* as the query against the *An. gambiae* PEST, M and S genome assemblies yielded no significant similarity*.* Additionally blastn searches with word size seven and e-value threshold of 1×10^-2^ using *sYG2* as the query against the PEST, M, and S trace files revealed no significant alignments. Tblastx searches with an e-value threshold of 1×10^-5^ yielded no significant similarity to the *An. gambiae* genome.

### Y gene finding in the African malaria mosquito *An. gambiae*

The AgamP3 genome assembly of the *An. gambiae* PEST strain was downloaded from VectorBase. The genome was divided into seven parts: the arms of the two autosomes, the X chromosome, fragments of the Y chromosome, and unmapped sequences referred to as UNKN. The repetitive sequences of the AgamP3 genome assembly were masked using RepeatMasker by VectorBase and indicated by lowercase nucleotides.

The repetitive sequences indicated by RepeatMasker were removed from the autosomes, X, Y, and UNKN sequences creating many smaller fragments. Sequences shorter than 250 bases were removed to mitigate false positives. The number and N50 size of the fragmented autosome, X, Y, and UNKN sequences were calculated (Additional file 5: Table S2). Illumina sequencing of male and virgin female *An. gambiae* was performed on pools of approximately 30 individuals from the G3 strain from the same colony [SRA: SRP014730] (Additional file 6: Table S3). Chromosome quotients were calculated for the fragmented *An. gambiae* autosomes, X, Y, and UNKN sequences using the Illumina sequence data mentioned above.

We were concerned that Sanger sequencing, which was used to sequence the *An. gambiae* genome, may be biased against the heterochromatic Y chromosome. We attempted to circumvent this bias by assembling our male G3 strain Illumina data and searching for Y sequences. We assembled the *An. gambiae* male Illumina sequence data using ABySS single-end assembly with the kmer setting 31 [41]. The number of contigs and the N50 contig size of the assembly were calculated (Additional file 5: Table S2). Since the N50 contig size was so short for this assembly, no further fragmentation was deemed necessary. Chromosome quotients were calculated for all the contigs in this assembly using the male and female *An. gambiae* Illumina data.

We searched for Y genes in the sequences with chromosome quotients less than 0.3 using the raw reads from *An. gambiae* transcriptome sequence data. Using blastn requiring 100 percent identity and an e-value less than 1×10^-5^ we compared the Y-linked contigs to transcriptome sequence data from adult male *An. gambiae* [SRA: SRP014756]. Using the same parameters, we also compared the UNKN sequences that we inferred to be Y-linked by their CQs to the same transcriptome data (Additional file 7: Table S4).

We searched for the *An. gambiae* Y chromosome genes *gYG1 and gYG2* in the *An. stephensi* genome using blastn and tblastx. Blastn searches with a word size of 7 and an e-value threshold of 1×10^-5^ using the sequence of *gYG1* and *gYG2* as a query yielded no similarity to the *An. stephensi* genome. Furthermore, using blastn we compared *gYG1* and *gYG2* to Illumina data from *An. stephensi* and we found no significant similarity using word size seven and an e-value threshold of 1×10^-2^. Tblastx searches with an e-value threshold of 1×10^-5^ only yielded significant alignments from a small repetitive part of *gYG1* to the *An. stephensi* genome.

### Molecular biology methods

Genomic DNA samples were isolated with Life Technologies DNAzol from male and virgin female mosquitoes from the Indian wild type strain of *An. stephensi* and the G3 strain of *An. gambiae.* Five male and female samples were prepared, each from five individuals. In the case of Y genes that have autosomal or X paralogs, primers for genomic DNA PCR were designed with the differences between the autosomal or X paralog and the Y sequence at the 3′ end of the primer. PCR was performed with Finnzymes Phire DNA Polymerase. RNA was isolated from embryos, larva, pupa, adult male, and adult female individuals with the Life Technologies mirVana RNA isolation kit using the total RNA isolation protocol. Complementary DNA was synthesized with Life Technologies SuperScript III RT. All primer sequences are available in the additional files (Additional file 8: Table S5). We used primer-sets that exhibited male-specific amplification to perform RT-PCR with the complementary DNA mentioned above as template with either Finnzymes Phire DNA Polymerase or TaKaRa rTaq. Rapid amplification of cDNA ends (RACE) was performed on *sYG1, gYG2*, and *gYG3* using the SMARTer RACE cDNA Amplification Kit. The resulting sequences were assembled into full-length transcripts, and verified by sequencing complementary DNA. Digital PCR was performed with a QX100 Droplet Digital PCR System from Bio-Rad, on male and female genomic DNA with a probe that would hybridize to both *sYG1* and *AsA-bbx*. A single copy autosomal gene, zeta DNA polymerase catalytic subunit, was used as the reference set as two copies per diploid genome. Chromosomal fluorescence *in situ* hybridization for *sYG1* was performed on mitotic and polytene chromosomes using the method described in [42-44]. Phylogenetic analysis was performed with MrBayes [45]. The alignment and parameters used to infer the phylogeny are provided in the additional files (Additional file 9).

## Competing interests

The authors declare that they have no competing interests.

## Authors’ contributions

ABH conceived and implemented the CQ method and discovered the Y genes, performed PCR, and drafted the manuscript. YQ performed PCR and RACE. VT, MS, and IS performed FISH. IS and MS helped with manuscript revision. ZT initiated and designed the project, performed evolutionary analysis, and contributed to Y gene discovery and the writing of the manuscript. All authors read and approved the final manuscript.

## Supplementary Material

Additional file 1: Figure S1 The calculation of chromosome quotients. Click here for file

Additional file 2: Figure S2 Autosome, X, and Y sequences differ in the number of alignments from male and female sequence data. Click here for file

Additional file 3 **Sequences referenced in the text including: the six Y genes, intergenic Y sequences tested for male-specific amplification, *An. stephensi *Y sequences, *An. gambiae *Y sequences, the autosomal homolog of *sYG1, *and the revised *An. stephensi *scaffold linking the autosomal homolog of *sYG3 *to a long scaffold.** Click here for file

Additional file 4: Table S1 Analysis of the male alignment threshold. Click here for file

Additional file 5: Table S2 Statistics from the fragmented genome assemblies. Click here for file

Additional file 6: Table S3 The coverage of the male and female sequence data used in the study. Click here for file

Additional file 7: Table S4 The number of reads and read length from the RNA-seq data used in the study. Click here for file

Additional file 8: Table S5 Sequences of the primers used in the study. Click here for file

Additional file 9 Alignment and parameters used to infer phylogeny. Click here for file
