# Supplementary material for: Six novel Y chromosome genes in Anopheles mosquitoes discovered by independently sequencing males and females
Source: BMC Genomics. 2013 Apr 23;14:273. doi: 10.1186/1471-2164-14-273 (PMC3660176; doi:10.1186/1471-2164-14-273)
Supplement: Additional file 1: Figure S1 — The calculation of chromosome quotients. [file 1471-2164-14-273-S1.pdf]

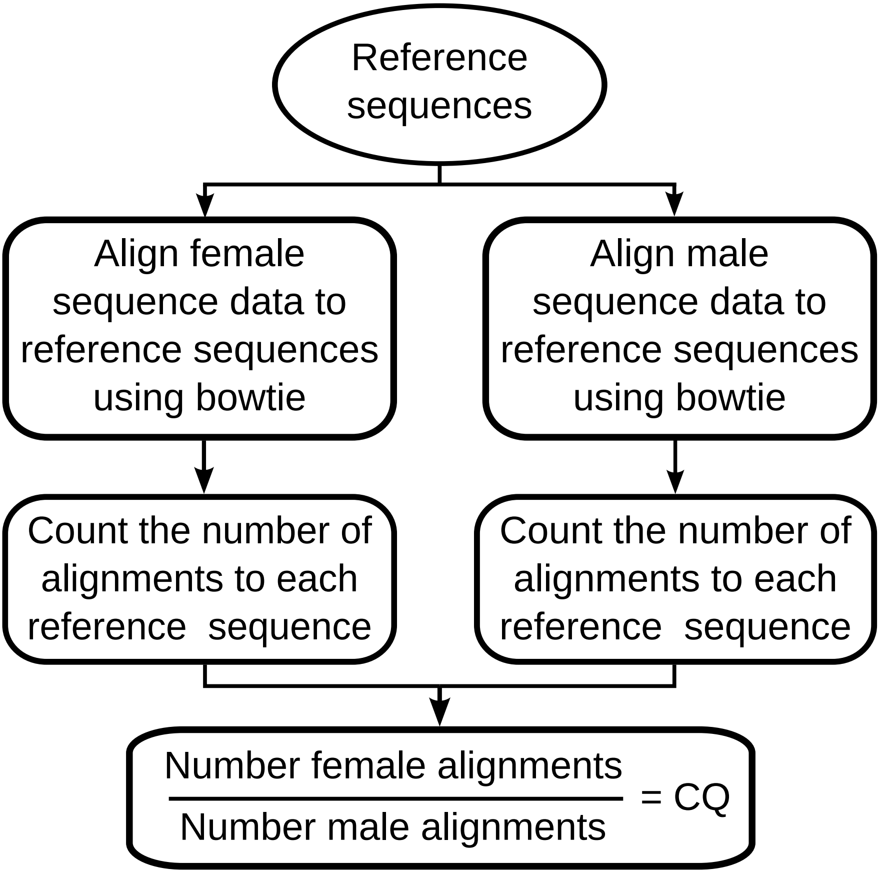

Additional file 1: Figure S1 - Calculating chromosome quotients

Chromosome quotients are calculated by aligning female and male sequence data to reference sequences using the ultra-fast short read aligner bowtie. For a read to be aligned to a reference sequence, it must have zero mismatches over the entire length of the read. Once the male and female data have been aligned to the reference sequences, the number of alignments from male and female data to each reference sequence is counted. Chromosome quotients are then calculated by dividing the number of female alignments by the number of male alignments. If known autosomal sequences are present, all the chromosome quotients can be normalized the the median chromosome quotient of the known autosomal sequences.
