# Supplementary material for: Six novel Y chromosome genes in Anopheles mosquitoes discovered by independently sequencing males and females
Source: BMC Genomics. 2013 Apr 23;14:273. doi: 10.1186/1471-2164-14-273 (PMC3660176; doi:10.1186/1471-2164-14-273)
Supplement: Additional file 2: Figure S2 — Autosome, X, and Y sequences differ in the number of alignments from male and female sequence data. [file 1471-2164-14-273-S2.pdf]

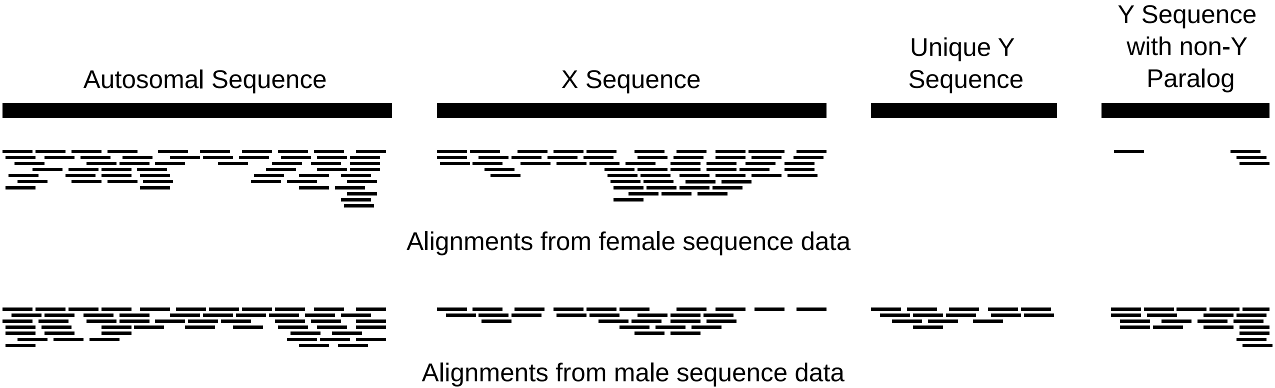

Additional file 2: Figure S2 - Alignments from male and female sequence data to autosomal, X and Y chromosome sequences

An approximation of alignments from male and female sequence data to demonstrate how Y chromosome sequences can be differentiated by their distinctive chromosome quotients. Males and females share the same complement of autosomes, so autosomal sequences are present in both the male and female sequence data in roughly the same quantities. Therefore, autosomal sequences have chromosome quotients distributed around one. Females have two X chromosomes while males only have one, so X chromosome sequences are present in female sequence data roughly twice as frequently as in male sequence data. Therefore, X chromosome sequences have chromosome quotients distributed around two. Unique Y chromosome sequences are only present in male sequence data, and therefore have chromosome quotients of zero. Repetitive or recently duplicated Y sequences may have a few alignments from female sequence data, but have more alignments from male sequence data.
