# Supplementary material for: Six novel Y chromosome genes in Anopheles mosquitoes discovered by independently sequencing males and females
Source: BMC Genomics. 2013 Apr 23;14:273. doi: 10.1186/1471-2164-14-273 (PMC3660176; doi:10.1186/1471-2164-14-273)
Supplement: Additional file 4: Table S1 — Analysis of the male alignment threshold. [file 1471-2164-14-273-S4.docx]

**Additional file 4: Table S1 - Male alignment threshold analysis**

| *H. sapiens* | | | |
| --- | --- | --- | --- |
| Threshold of Male Alignments | Total Sequences Having Threshold | False Positives | Percent False Positives |
| 1 | 1,470,530 | 2,278 | 0.15491 |
| 5 | 1,403,318 | 1,590 | 0.113303 |
| 10 | 1,206,908 | 729 | 0.060402 |
| 15 | 986,261 | 287 | 0.0291 |
| 20 | 803,633 | 124 | 0.01543 |
| 25 | 663,825 | 82 | 0.012353 |
| 30 | 556,287 | 68 | 0.012224 |
| 35 | 472,830 | 54 | 0.011421 |
| 40 | 407,355 | 46 | 0.011292 |
| 45 | 356,123 | 43 | 0.012074 |
| 50 | 314,977 | 41 | 0.013017 |

| *D. melanogaster* | | | |
| --- | --- | --- | --- |
| Threshold of Male Alignments | Total Sequences Having Threshold | False Positives | Percent False Positives |
| 1 | 4,2618 | 3 | 0.00704 |
| 5 | 4,2616 | 1 | 0.00235 |
| 10 | 4,2614 | 0 | 0 |
| 15 | 4,2614 | 0 | 0 |
| 20 | 4,2614 | 0 | 0 |
| 25 | 4,2614 | 0 | 0 |
| 30 | 4,2613 | 0 | 0 |
| 35 | 4,2613 | 0 | 0 |
| 40 | 4,2613 | 0 | 0 |
| 45 | 4,2612 | 0 | 0 |
| 50 | 4,2610 | 0 | 0 |

| *An. gambiae* | | | |
| --- | --- | --- | --- |
| Threshold of Male Alignments | Total Sequences Having Threshold | False Positives | Percent False Positives |
| 1 | 159,278 | 4,393 | 2.75807 |
| 5 | 152,493 | 3,331 | 2.18436 |
| 10 | 143,336 | 2,395 | 1.6709 |
| 15 | 134,060 | 1,643 | 1.22557 |
| 20 | 125,508 | 1,187 | 0.94576 |
| 25 | 117,595 | 870 | 0.73983 |
| 30 | 110,247 | 666 | 0.6041 |
| 35 | 103,744 | 510 | 0.49159 |
| 40 | 97,758 | 388 | 0.3969 |
| 45 | 92,178 | 319 | 0.34607 |
| 50 | 87,088 | 245 | 0.28132 |

The threshold is the minimum number of alignments from male sequence data. A higher threshold reduces the number of false positives, but also reduces the total number of sequences with the requisite number of alignments. We balance the rate of false positives with the total number of sequences with the requisite alignments by choosing a threshold of 30. The threshold is flexible, and if more confidence is required, a higher threshold can be used. If a low rate of false negatives is needed a lower threshold can be used.
