# Supplementary material for: Six novel Y chromosome genes in Anopheles mosquitoes discovered by independently sequencing males and females
Source: BMC Genomics. 2013 Apr 23;14:273. doi: 10.1186/1471-2164-14-273 (PMC3660176; doi:10.1186/1471-2164-14-273)
Supplement: Additional file 5: Table S2 — Statistics from the fragmented genome assemblies. [file 1471-2164-14-273-S5.docx]

**Additional File 5: Table S2 - Number of sequences and N50 size of fragmented genomes**

| Sequences | Number of Sequences | N50 size |
| --- | --- | --- |
| *H. sapiens* autosomes | 1,477,828 | 1,001 |
| *H. sapiens* X | 65,862 | 969 |
| *H. sapiens* Y | 10,159 | 1,022 |
| *D. melanogaster* autosomes | 42,625 | 3,334 |
| *D. melanogaster* X | 14,593 | 1,915 |
| *D. melanogast*er Y | 106 | 973 |
| *An. gambiae* PEST autosomes | 163,892 | 1,385 |
| *An. gambiae* PEST X | 21,068 | 801 |
| *An. gambiae* PEST Y | 42 | 778 |
| *An. gambiae* PEST UNKN | 10,453 | 3,112 |
| *An. gambiae* G3 Illumina assembly | 214,099 | 639 |
| *An. stephensi* autosomes | 2,354 | 27,737 |
| *An. stephensi* X | 188 | 17,817 |
| *An. stephensi* whole genome | 113,570 | 4,427 |

The number of sequences and N50 size of sequences resulting from fragmenting chromosomes by removing repeats indicated by RepeatMasker. Fragments less than 250 bases were removed to prevent false positives from very short sequences.
