# Supplementary material for: Six novel Y chromosome genes in Anopheles mosquitoes discovered by independently sequencing males and females
Source: BMC Genomics. 2013 Apr 23;14:273. doi: 10.1186/1471-2164-14-273 (PMC3660176; doi:10.1186/1471-2164-14-273)
Supplement: Additional file 6: Table S3 — The coverage of the male and female sequence data used in the study. [file 1471-2164-14-273-S6.docx]

**Additional file 6: Table S3 - Coverage Table**

| Species | Sex | Source | Reads | Read Length | Coverage |
| --- | --- | --- | --- | --- | --- |
| *H. sapiens* | Female | HG00235 | 838,286,979 | 50 | 14x |
|  | Male | HG00234 | 690,062,773 | 50 | 11x |
| *D. melanogaster* | Female | SRP007888 | 400,368,801 | 36 | 82x |
|  | Male | SRP007888 | 391,872,371 | 36 | 80x |
| *An. stephensi* | Female | SRR643416 | 36,083,333 | 84 | 12x |
|  | Male | SRR643415 | 35,103,726 | 84 | 12x |
| *An. gambiae* | Female | SRR534286 | 114,736,126 | 76 | 31x |
|  | Male | SRR534285 | 103,809,892 | 77 | 29x |

The coverage of the male and female sequence data for the four species analyzed in this study.
