# Supplementary material for: Six novel Y chromosome genes in Anopheles mosquitoes discovered by independently sequencing males and females
Source: BMC Genomics. 2013 Apr 23;14:273. doi: 10.1186/1471-2164-14-273 (PMC3660176; doi:10.1186/1471-2164-14-273)
Supplement: Additional file 7: Table S4 — The number of reads and read length from the RNA-seq data used in the study. [file 1471-2164-14-273-S7.docx]

**Additional file 7: Table S4 - The number of reads from the RNA-seq data used in this study**

| Time point | Reads | Read length |
| --- | --- | --- |
| *An. stephensi* RNA-seq | | |
| 0-1 hour embryo | 5,524,113 | 41 |
| 2-4 hour embryo | 5,210,129 | 41 |
| 4-8 hour embryo | 8,270,650 | 39 |
| 8-12 hour embryo | 14,234,461 | 38 |
| Larva | 13,962,583 | 41 |
| Pupa | 9,800,361 | 41 |
| Adult Female | 14,085,091 | 41 |
| Adult Male | 16,437,913 | 41 |
| *An. gambiae* RNA-seq | | |
| Adult Male | 25,771,954 | 50 |

The number of reads and read length from the *An. stephensi* [SRA: SRP013839] and *An. gambiae* [SRA: SRP014756] RNA-seq data.
