# Supplementary material for: Six novel Y chromosome genes in Anopheles mosquitoes discovered by independently sequencing males and females
Source: BMC Genomics. 2013 Apr 23;14:273. doi: 10.1186/1471-2164-14-273 (PMC3660176; doi:10.1186/1471-2164-14-273)
Supplement: Additional file 8: Table S5 — Sequences of the primers used in the study. [file 1471-2164-14-273-S8.doc]

**Additional file 8: Table S5 - Primer Sequences**

| **Sequence Name** | **Primer Sequences** |
| --- | --- |
| **sYG1** | F: ACGAAAGTGCCGCTTGTAG  R: CCTGCACACTCGGGTACATA |
| **sYG2** | F: TGCCGGACATGACATTTG  R: TCAATGCGAACAGAAGGCTAA |
| **sYG3** | F: CTGGTGGTTTGAGCTTCTCT  R: CCAAACCAGAAACCGTAACC |
| **gYG1** | F: TTAGTGAGCTGCCGCATTATT  R: TTGGCTTCAACTCGACCTCT |
| **gYG2** | F: CGCGCACTTGTTTATACTGTTAC  R: CGAAAGGGAAAGTTACGAGC |
| **gYG3** | F: TCAGGTTTCGATCGGTCAG  R: GGATCTCGCATTTCTTTCAGAC |
| ***An. stephensi* Y sequence 1** | F: GCGATCGAATAAAACCGGT  R: GGCGATATGGAAGGCTTAAAC |
| ***An. stephensi* Y sequence 2** | F: TTCTGCATATTTCGCGGG  R: GCCAAACGGCTTCGATACT |
| ***An. stephensi* Y sequence 3** | F: CTCACCGCATACACACTTTTCA  R: TTTGCCTTCCTGCAGTGACT |
| ***An. stephensi* Y sequence 4** | F: GATGTGCCTTAATTCCATTTCG  R: GACGGAGTTACGCACACCTTA |
| ***An. stephensi* Y sequence 5** | F: GACCCCATATTAGACAGCGATTT  R: TTTACGACGCTACGCCG |
| ***An. gambiae* Y sequence 1** | F: TTTGCAGCTCGCTTTCG  R: GAATCATCTCAATTTTCGCC |
| ***sYG1* Digital PCR Primer** | F: ACACATCACAGCGCAACGA  R: CGGCCGGCGTTTGG  Probe: CACGTGCCAGCAGCACATGGT |
| **Auto Reference Digital PCR Primer** | F: GTCCCTCGAGAATTTTCGTAAACTG  R: TTGCGCATACTGTTCGAAACTTG  Probe: CGACAAGTGTACTGCCGCACCTTC |

Primer sequences used to amplify the Y genes and Y sequences along with the probes and autosomal reference primers used for digital PCR.
